# Supplementary figures and images for: The Ubiquitin Proteasome System Is a Key Regulator of Pluripotent Stem Cell Survival and Motor Neuron Differentiation
Source: Cells. 2019 Jun 13;8(6):581. doi: 10.3390/cells8060581 (PMC6627164; doi:10.3390/cells8060581)

A

Synaptic

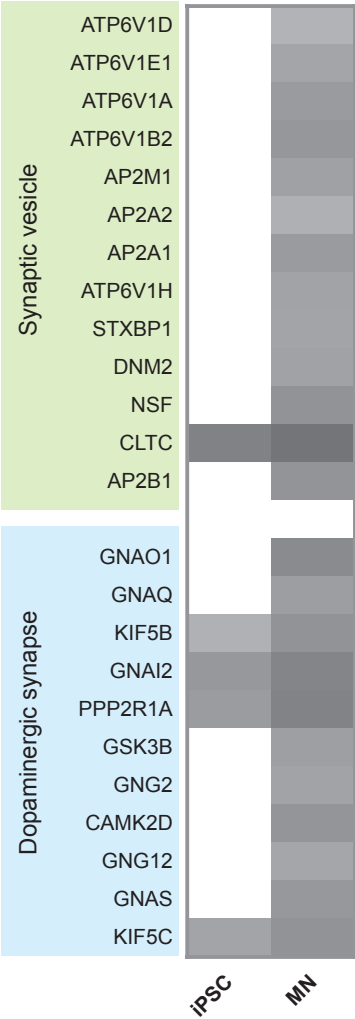

B

Metabolism

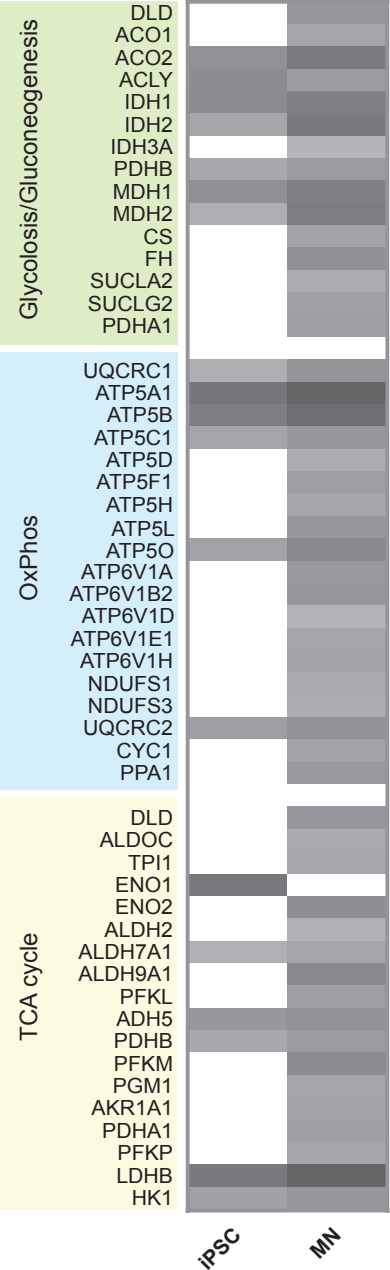

C

Cell/matrix interaction

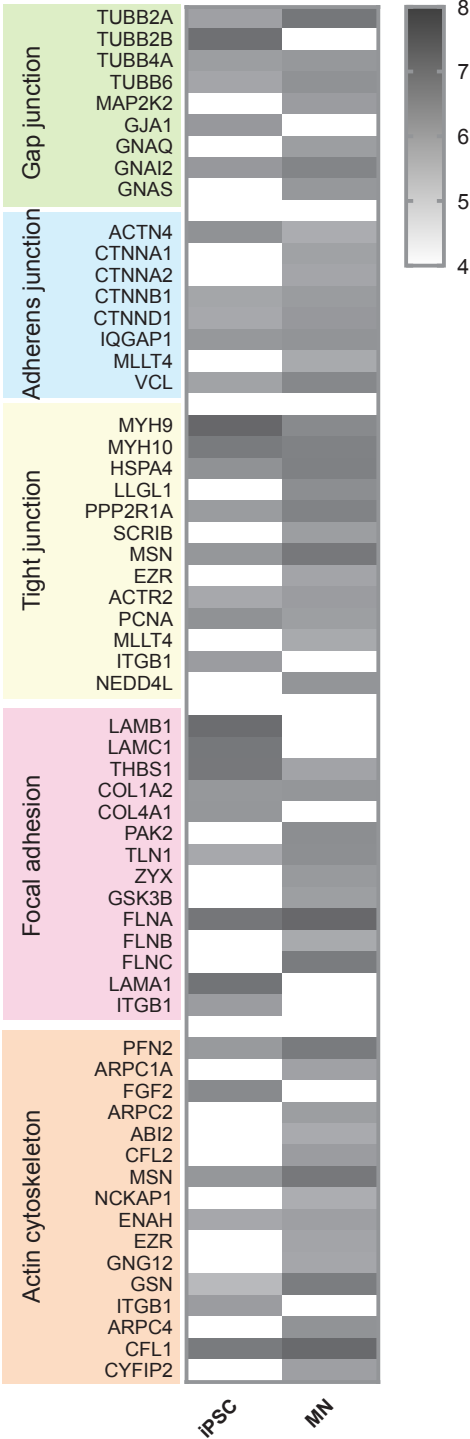

D

Spliceosome

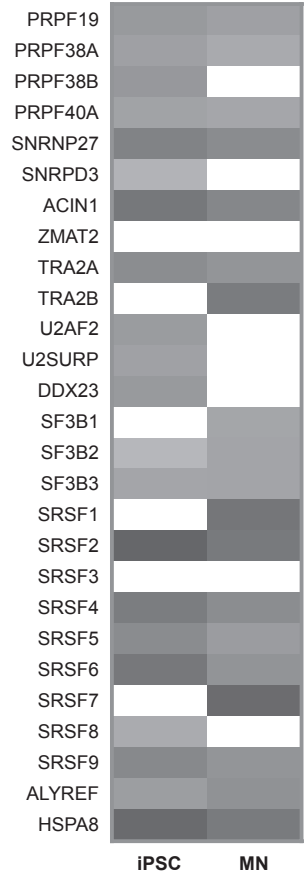

Supplement: Supplementary file 1 [file cells-08-00581-s001.pdf]
